# Women in chemistry: Q&A with Professor Carolina Horta Andrade

**DOI:** 10.1038/s42004-025-01452-y

**Published:** 2025-02-22

## Abstract

Carolina Horta Andrade is an Associate Professor of Medicinal Chemistry at the Faculty of Pharmacy of Federal University of Goiás, Brazil, and head of LabMol – Laboratory for Molecular Modeling and Drug Design.

Carolina received her degree in Pharmacy from the Federal University of Goias (UFG) and a Ph.D. in Drugs and Medicines from University of Sao Paulo (USP, Brazil). In addition to her role at the Faculty of Pharmacy of Federal University of Goiás, since 2021 she is head of pharmaceutical projects at the Center for Excellence in Artificial Intelligence (CEIA) and a principal investigator of the Advanced Knowledge Center for Immersive Technologies (AKCIT), UFG. She is also one of the coordinators of the Center for Research and Advancement on Fragments and Molecular Targets (CRAFT).

**Figure:**
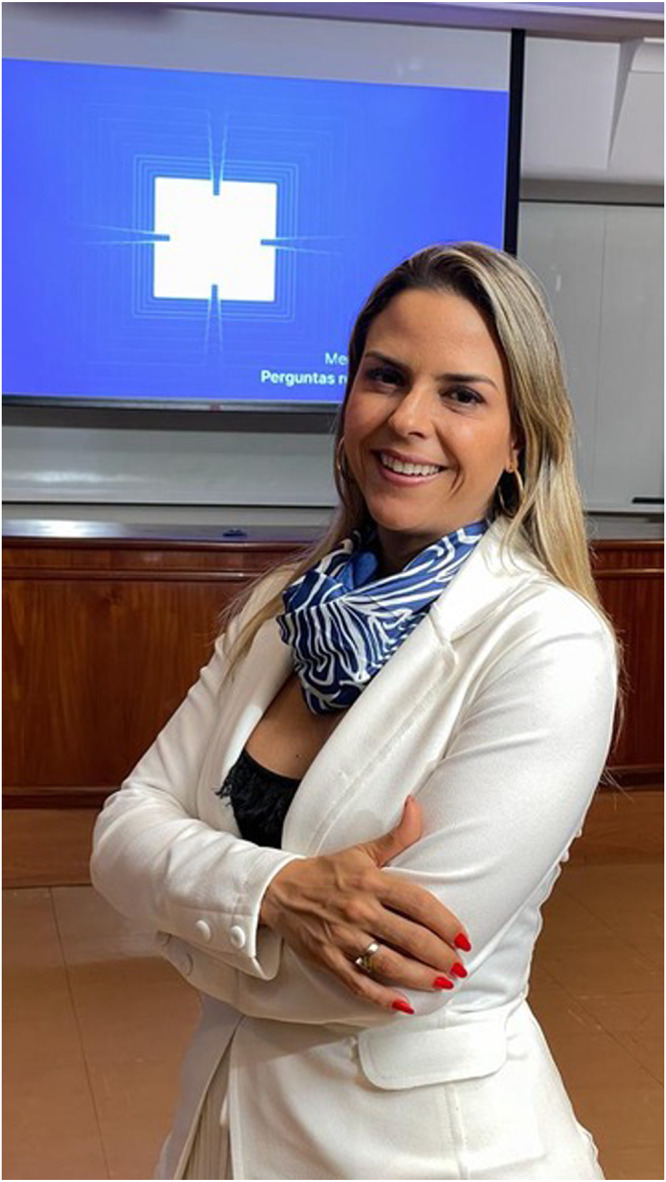
Murillo Guedes

Her research focuses on computer-assisted and artificial intelligence-oriented drug design, aiming at discovering new drug candidates for neglected and emerging diseases. Her research also focuses on the development of AI tools and QSAR models for toxicological research and risk assessment. Her major software tools and models developed are publicly available in LabMol web portal www.labmol.com.br. She has supervised 19 master’s and 11 doctoral theses. In 2014, she was awarded the For Women in Science award from L’Oréal-UNESCO and the Brazilian Academy of Sciences in Brazil, and in 2015 the International Rising Talents award from L’Oréal—UNESCO in France. In 2022, the American Chemical Society (ACS) and the Brazilian Society of Chemistry (SBQ) awarded her with the Brazilian Women in Chemistry Award. In 2024, she received the Cavaliere dell’Ordine della Stella d’Italia” medal by the President of the Italian Republic, to celebrate excellence in science and promotion of inter-university cooperation between Italy and Brazil. She was elected to the Young Academy of the Brazilian Academy of Sciences (2016-2021), and to the Global Young Academy (GYA, 2020-2023).

She served as Associate Editor for the Artificial Intelligence in the Life Sciences (2021-2022) and as Editorial Board Member of Communications Chemistry (2020–2022). She currently serves as Senior Editor for ACS Omega and is member of the Editorial Advisory Boards for the Journal of Medicinal Chemistry, ACS Medicinal Chemistry Letters, Journal of Chemical Information and Modelling and ChemMedChem. She has published over 150 ISI papers, 9 book chapters, filed 2 patents, and developed 4 technological products (software), with more than 6436 Google Scholar citations resulting in an h-index of 41. In 2023 and 2024, she was ranked the top 2% most cited scientists in the world (Elsevier/Stanford University).

Why did you choose to be a scientist?

I chose to become a scientist because of a gradual realization of my passion for research and inquiry, which began during my undergraduate studies in Pharmacy. I have always been fascinated by science, natural phenomena, and biological processes, fueled by my curiosity from a young age.

As a child, I enjoyed watching TV shows like “Beakman’s World” and participated in science fairs throughout primary and secondary school. These experiences provided me with a closer glimpse of what it means to be a scientist; in those moments, we considered ourselves “junior scientists”, and they inspired me to follow this path. Additionally, through my participation in Scientific Initiation and the development of my Final Graduation Project (FGP), I discovered a profound interest in the academic field, particularly in medicinal chemistry. This discipline captivates me for its potential to create tangible improvements in health and well-being.

My paternal grandfather has also played an inspiring role in my journey, albeit unconsciously. Moacyr Duval Andrade was a Full Professor at UFMG. Unfortunately, he passed away long before I was born, and I never had the privilege of knowing him. However, my father and uncles often share inspiring stories about his life. He graduated from the Federal University of Ouro Preto and received the Golden Medal, awarded to the top student in the Civil Engineering School. He was fluent in nine languages and held international respect in the fields of Civil Engineering and Electrical Engineering.

Ultimately, my journey into science feels organic, as I have been able to merge my interests with meaningful research. Becoming a scientist enables me to explore the unknown, contribute to advancements in health, and engage in lifelong learning—all of which I find deeply rewarding.

What scientific development are you currently most excited about?

I am currently leading a groundbreaking and exciting initiative known as SOFIA, which stands for Sensorial Olfactory Framework Immersive with AI. This research and development project is part of the Advanced Knowledge Center for Immersive Technologies (AKCIT, https://akcit.ufg.br/) which aims to position Brazil as a leader in the fields of artificial intelligence and virtual reality. SOFIA project resonates deeply with me because olfaction is a complex sensation arising from the interaction of small chemical molecules, known as odorants, with olfactory receptors. This intricate interaction is at the heart of our research, as we strive to understand how a single olfactory molecule can interact with multiple receptors simultaneously, including the orphan olfactory receptors that do not interact with known odorants.

The primary goal of the SOFIA project is to apply artificial intelligence to develop advanced computational models designed for immersive olfactory experiences. We aim to seamlessly integrate these models into multi-sensory environments using innovative devices that generate precise directional stimuli, maintaining the appropriate intensity while allowing for rapid neutralization of odors.

Our exploration is enhanced by employing techniques that merge AI with neuroscience to study these complex interaction networks. The intersection of technology and sensory experience not only opens up new avenues in virtual reality but also has the potential to significantly enhance user engagement and emotional impact. This potential for innovative application, which exemplifies the beauty of chemistry, is incredibly inspiring to me.

What direction do you think your research field should go in?

I believe that the future direction of my research field should heavily embrace the advancements in artificial intelligence technologies, particularly in virtual reality (VR). My current work focuses on developing AI models that significantly enhance the immersive experience, with applications spanning various fields, including education, healthcare and cosmetology. This area of research is on the cutting edge, as only a small number of scientists globally are exploring these innovative intersections, and we are optimistic about bringing a new product to fruition soon.

Additionally, in the field of drug discovery, I see tremendous potential for AI to transform traditional methodologies. AI is increasingly being utilized to support decision-making processes and expedite the identification of high-quality hits and leads. As generative design, large language models and other AI methods continue to evolve, I anticipate that we will see a new wave of drugs enter the market, developed with the aid of these advanced technologies. The integration of AI into both VR and drug discovery will not only enhance user experiences but also significantly improve efficiency and outcomes across diverse applications.

What aspects of your research do you find most (and least) exciting or rewarding?

I find the most stimulating aspect of my research to be the drive to develop medications for the thousands of individuals suffering from diseases that currently have no effective treatment. The opportunity to contribute to improving lives is incredibly motivating. Additionally, sharing my knowledge and experiences and teaching others is both inspiring and rewarding. However, there are moments of frustration when our efforts go unrecognized by society and government, which can make the journey challenging. Nonetheless, my commitment to advancing science remains unwavering.

How can young women in the field be supported to become established scientists?

I encourage young women in science to never give up and to always have confidence in themselves. It’s essential to pursue your dreams, even when it feels like the universe is conspiring against you. If you’re passionate about a scientific career, move forward with dedication and determination. Being a woman should never be seen as a barrier to success in the scientific field; we have the strength and intelligence to overcome challenges and achieve our goals.

It’s also important to seek out strong mentors and attend reputable universities that support your aspirations. Building networks and establishing connections within the scientific community can be incredibly beneficial. Create support systems among your peers and colleagues. Ensure your presence is known—don’t hold back and don’t be shy! Your voice and contributions are valuable, and it’s vital to assert yourself in this field. We all need support—a strong network that includes family, friends, and colleagues. Take the initiative to build your own support network!

Could you describe a memorable moment in your career where being a woman made a significant difference, possibly even steered your path in a certain direction?

One of the most memorable moments in my career was receiving the Brazilian Women in Chemistry Award in 2022, presented by the American Chemical Society and the Brazilian Chemical Society. This recognition came at a pivotal time, right after I returned from maternity leave with my second child. The COVID-19 pandemic had caused me (probably everyone) to seriously reevaluate my future and question whether I wanted to continue living a life fully dedicated to my work.

Becoming a mother inevitably shifts priorities, and I realized that I would never be the same Carolina I was before having children. Despite this, my passion for science and my work remained strong. When I received the award during the first in-person Brazilian Chemical Society National Meeting after the pandemic, I felt a renewed sense of vitality. It was a reminder to myself that I could not set aside my love for science!

Today, my greatest challenge is finding a balance among my various roles: as a mother, scientist, wife, and daughter. While my priorities have changed, the things I am passionate about have not diminished. This experience has reinforced the importance of resilience and adaptability, not just for me but for all women striving to pursue their careers while navigating the complexities of life. And again, we need support to keep going!

Are you or have you been supported by a mentor? What was the best advice you received?

Yes, for sure! I have been fortunate to receive support from three influential scientists in my life: my three advisors, two of whom are women—Prof. Elizabeth Ferreira from USP and Dr. Kerly Pasqualoto—along with my co-advisor in the United States, Prof. Anton Hopfinger. Both women have served as inspiring role models and leaders in their fields. Prof. Beth, always poised and articulate, continues to inspire me to this day—she is truly an incredible woman! My dear Kerly was always ready to help, even late at night and on weekends. I would go to her house with my desktop computer, and we would spend hours installing programs and running calculations on Linux. I learned so much from her!

Professor Tony, who sadly passed away in 2018, was a pivotal figure in my career. I spent about 9 months in Albuquerque, New Mexico, working with him at the University of New Mexico, where he served as a Distinguished Research Professor of Pharmacy. Previously, he had an illustrious career at UIC in Chicago as a Professor Emeritus of Medicinal Chemistry and Pharmacognosy at the University of Illinois. Additionally, he was the Founder, Chief Technical Officer, and Secretary of The Chem21 Group.

Professor Tony developed several cheminformatics methods and QSAR software, including the innovative 4D-QSAR methodology. His groundbreaking contributions led to the development of Aspartame, one of the world’s leading artificial sweeteners, as well as Donezepil (Aricept®), a blockbuster drug for treating early-stage Alzheimer’s disease. His research also played a crucial role in the creation of Celecoxib (Celebrex®), which is used for treating pain and inflammatory diseases.

Prof. Tony was not only brilliant but also incredibly approachable and humble. He consistently provided me with innovative ideas to solve my problems and offered generous praise. I will never forget the words in his letter of recommendation for me: “Simply put, Carolina is one of the best graduate students (from more than 85) who have studied with me. I am particularly impressed with her fortitude, creativity, and self-reliance.”

By the end of my Ph.D. studies, we published two articles in internationally recognized journals, with him naming me as the corresponding author^1,2^. This recognition was a game-changer for my career! Shortly after defending my PhD, I was invited to speak at several international conferences about my work—an opportunity that marked my transition to a professional scientist. I owe a great deal to my mentors for their invaluable support!

Since 2021, I am fortunate to work with and learn from three inspiring leaders: Professors Telma Soares, Anderson Soares, and Arlindo Galvão, the CEO and leaders from CEIA (Excellence Center of Artificial Intelligence, https://ceia.ufg.br/) and AKCIT. They have taught me invaluable lessons about translating our research into practical solutions for businesses, emphasizing the importance of adding business value to our work and adopting a more entrepreneurial perspective rather than a purely academic one. I learn from them daily how to effectively engage with companies and how to better promote and sell our ideas and innovations.

How can publishers, editors, funders, and conference organizers better support women scientists?

First and foremost, achieving a balanced representation of men and women in editorial positions is essential. Also, it’s very important to ensure gender parity among reviewers, awardees of grants, and leadership roles within academic publishing. Women must be afforded the same opportunities and conditions as their male counterparts to advance in their careers.

Additionally, motherhood should not be perceived as a hindrance. It is crucial that governments provide support through improved policies that promote shared parental leave with fathers to foster a more equitable division of responsibilities. This would not only empower women but also encourage a culture that values family commitments equally among all genders.

Furthermore, conferences should offer child-friendly spaces and activities at little to no additional cost, allowing mothers to attend while caring for their children. By integrating these family-oriented solutions, we can create an inclusive environment that enables women scientists to participate fully in their professional communities.

Overall, a multi-faceted approach that includes equitable representation, supportive parental leave policies, and family-friendly conference environments will significantly contribute to empowering women in science and ensuring their contributions are recognized and valued.

How can individual scientists support and celebrate their women colleagues?

Recognize and appreciate the contributions of fellow women scientists! Elevate their achievements and engage in meaningful conversations with them to celebrate your successes together. It’s essential that we unite rather than distance ourselves from one another. I believe this is one of the primary barriers we face.

While men often support and collaborate with one another, women, in general, tend to be less connected due to socio-cultural factors. We must work to change this narrative. By fostering a culture of solidarity and collaboration among women in science, we can create a supportive community that empowers each other.

Encourage one another, share resources, and celebrate each other’s accomplishments openly. Let’s build a network of support that champions women’s achievements and fosters a sense of belonging in the scientific community. Together, we can create lasting change and help each other thrive.

*This interview was conducted by the editors of Communications Chemistry*.
